# Novel FHL1 Mutation Associated With Reducing Body Myopathy

**DOI:** 10.7759/cureus.109269

**Published:** 2026-05-20

**Authors:** Kyle W Ruffing, Marie Rivera-Zengotita, Lee Kugelmann, Morgan Jordan

**Affiliations:** 1 Neurology, University of Florida, Gainesville, USA; 2 Pathology, University of Florida, Gainesville, USA; 3 Neurology, Cleveland Clinic, Port St. Lucie, USA

**Keywords:** adult-onset myopathy, electron microscopy, fhl1, muscle biopsy, novel variant, reducing body myopathy

## Abstract

Reducing body myopathy (RBM) is a rare X-linked myopathy caused by mutations in the FHL1 gene and characterized by intracytoplasmic aggregates that reduce menadione nitroblue tetrazolium. We report a 45-year-old female presenting with progressive proximal weakness of unknown etiology. Prior muscle biopsy and genetic testing were non-diagnostic. By repeating her muscle biopsy in a different muscle, we were able to correlate the biopsy findings with her genetic variant previously described as a variant of unknown significance. This is the first reported case of possible RBM associated with FHL1 c.401A>C (p.Gln134Pro). The case expands the genotypic spectrum of RBM and underscores the diagnostic value of repeated muscle biopsy.

## Introduction

Reducing body myopathy (RBM) is a rare X-linked neuromuscular disorder first described by Brooke and Neville in 1972 [1]. It is characterized by distinctive intracytoplasmic inclusions, known as reducing bodies, that strongly stain with menadione-nitroblue tetrazolium (NBT) due to their ability to reduce NBT [2,3]. These aggregates represent abnormal protein accumulation within muscle fibers and are considered a hallmark of the disease.

RBM is caused by pathogenic variants in the FHL1 gene, which encodes four-and-a-half LIM domain protein 1, a structural and signaling protein expressed in skeletal and cardiac muscle [4]. Mutations in FHL1 have been associated with a spectrum of X-linked myopathies, including RBM, X-linked myopathy with postural muscle atrophy, and scapuloperoneal myopathy [5]. Clinical phenotypes range from severe, early-onset forms with rapid progression to milder, adult-onset presentations that may remain stable for years, particularly in heterozygous females due to variable X-inactivation [6]. Diagnosis may be challenging due to patchy histopathological involvement and phenotypic overlap with other myopathies, often requiring repeat biopsy or multimodal correlation. Cardiac involvement and respiratory compromise have been reported in some cases, underscoring the importance of early recognition and multidisciplinary management [7].

To date, more than 40 distinct FHL1 mutations have been identified, most of which cluster within LIM domains critical for protein-protein interactions [8]. Here, we report a novel FHL1 mutation that may be associated with RBM in an adult female.

## Case presentation

A 45-year-old woman presented with a seven-year history of progressive, asymmetric weakness predominantly affecting the proximal muscles of all limbs. She denied dysphagia, sensory symptoms, bulbar involvement, or respiratory difficulties. There was no family history of neuromuscular disorders. Previous treatment with prednisone had yielded no clinical improvement.

On neurological examination, muscle tone was normal, but there was a noticeable reduction in muscle bulk, particularly in proximal regions, with asymmetric involvement. Weakness was more pronounced proximally than distally, and right scapular winging was evident. Deep tendon reflexes were absent throughout. Sensory testing revealed intact function across all modalities.

A prior biopsy of the right deltoid muscle demonstrated nonspecific inflammatory infiltrates surrounding necrotic fibers within an end-stage muscle architecture. Electrodiagnostic studies revealed muscle membrane irritability, myopathic motor unit potentials, and early recruitment patterns on needle EMG (Table 1). EMG findings are consistent with a proximal, patchy myopathic process. 

**Table 1:** Electromyography summary table: concentric needle examination findings MUAP: motor unit action potentials, IA: insertional activity, PP: polyphasic motor units, N: normal, R: right

	Spontaneous activity			MUAP			Recruitment
Muscle	IA	Fibrillations	Fasciculations	Amplitude	Duration	PP	Pattern
R. deltoid	N	3+	None	1+	1+	2+	Reduced
R. triceps brachii	N	4+	None	1-	N	N	Reduced
R. biceps brachii	2-	None	None	2-	2-	3+	2+
R. extensor digitorum communis	N	3+	None	N	2+	3+	Discrete
R. first dorsal interosseous	N	2+	None	N	N	1+	Reduced
R. infraspinatus	N	3+	None	2-	2-	2+	2+
R. trapezius (upper)	N	3+	None	1-	N	N	1+
R. tensor fasciae latae	N	None	None				Cannot reach
R. tibialis anterior	N	None	None	N	N	N	N

Laboratory testing showed elevated creatine kinase 508 U/L (reference 30-240 U/L). Myositis panel was negative.

Muscle biopsy

**Figure 1:**
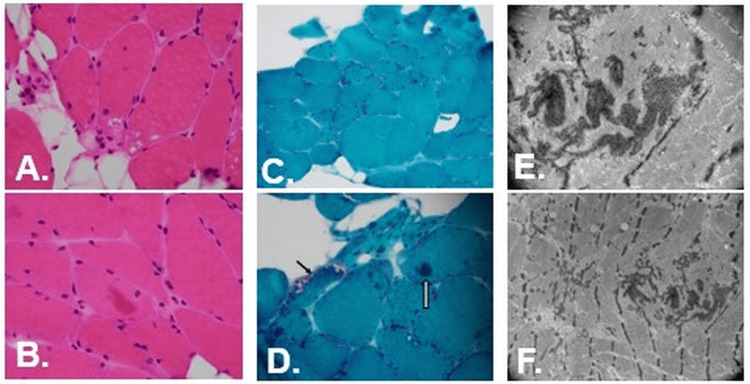
Histopathology of the right rectus femoris muscle biopsy (panels A–F). A-B: Hematoxylin and eosin (H&E)-stained sections showing myofibers with vacuoles (A) and dark eosinophilic sarcoplasmic inclusions (B). C-D: Gomori trichrome with ragged red-like fibers (C) and rimmed vacuole-like structures (black arrow) with dark red/green sarcoplasmic inclusions (open arrow) (D). E-F: Electron microscopy demonstrating sarcoplasmic aggregates of granular tubulo-filamentous material (E) and areas of myofibrillar architectural abnormalities (F).

Hematoxylin and eosin (H&E) showed vacuoles and eosinophilic inclusions; Gomori trichrome revealed ragged red-like fibers and rimmed vacuole-like structures; EM demonstrated tubulo-filamentous aggregates (Figure 1).

Genetic Testing

The invitae panel identified FHL1 c.401A>C (p.Gln134Pro), predicted deleterious by multiple in silico models (PP3), and lies within a conserved LIM domain critical for protein interactions. The variant is not reported in population databases (PM2). There are no prior definitive reports establishing pathogenicity, and the variant is therefore classified as a variant of uncertain significance (VUS). 

Alternative diagnoses, including inflammatory myopathy and sporadic nemaline myopathy, were considered but were not supported by serological testing, lack of treatment response, and pathological findings.

## Discussion

The first deltoid biopsy was insufficient to establish a diagnosis, likely due to the focal and asymmetric histopathological involvement typical of RBM [5,6]. Previous reports have emphasized that reducing bodies - as well as other FHL1-related pathological features - may be missed in early or limited tissue samples because of patchy distribution within affected muscles [5,6]. Evidence suggests that reducing body aggregates that accumulate and become more abundant over time, paralleling increasing clinical severity [5,6]. Adult-onset RBM typically manifests in the third to fourth decades of life, with asymmetric, predominantly proximal weakness - often involving scapuloperoneal muscles - and invariably progressive course [7]. Molecular insights support a common pathogenic mechanism across FHL1 mutations resulting in RBM and related conditions. In vitro expression of RBM-linked FHL1 mutants yields menadione-NBT-positive protein aggregates in myotubes and impairs myoblast differentiation, a dominant-negative or toxic gain-of-function effect [8].

To date, more than 40 FHL1 mutations have been identified, most clustering within LIM domains critical for protein-protein interactions [4,7]. The patient's phenotype - adult-onset, asymmetric proximal weakness in a female - is consistent with reported manifestations in heterozygous carriers [7]. Variable expression may reflect skewed X-inactivation, although this was not evaluated. The FHL1 c.401A>C (p.Gln134Pro) variant lies within a LIM domain, and similar domain variants have been implicated in RBM. However, in the absence of segregation analysis, functional validation, or population-level evidence, pathogenicity cannot be definitively established. In accordance with ACMG/AMP criteria, this variant remains classified as a VUS. Limitations include the lack of familial testing, the absence of X-inactivation studies, and the lack of direct functional assays.

Therefore, this report should be interpreted as demonstrating a clinicopathologic association rather than a confirmed causal relationship. This case illustrates the importance, as well as limitations, of integrating genetic and pathological findings without overinterpreting causality. Despite these limitations, this case expands the known clinical and pathological spectrum of FHL1-associated myopathies and reinforces the importance of correlating genetic findings with evolving biopsy features. 

## Conclusions

Women who are carriers of X-linked hereditary myopathies are frequently misdiagnosed, often due to variable clinical expression and incomplete penetrance. Mutations in FHL1 can present with diverse neuromuscular phenotypes, including adult-onset, sporadic cases characterized by asymmetric, progressive muscle weakness. We describe a possible association between the FHL1 c.401A>C (p.Gln134Pro) variant and RBM in an adult female. Diagnosis was achieved through an integrated approach combining clinical evaluation, electrodiagnostic studies, histopathology, and genetic analysis. Despite an initial biopsy that was non-diagnostic and a genetic variant initially classified as of uncertain significance, the repeat muscle biopsy revealed characteristic reducing bodies, enabling a definitive diagnosis. However, pathogenicity remains unproven, and further studies, including familial and functional analyses, are necessary to confirm this relationship. This case underscores the importance of correlating genetic findings with evolving pathological features to improve diagnostic accuracy.
